# Reply to: ‘Transforming cardiology with AI: the eko CORE 500 digital stethoscope’

**DOI:** 10.1007/s12471-025-01974-z

**Published:** 2025-08-01

**Authors:** H. Nathoe, Pim van der Harst

**Affiliations:** Department of Cardiology, Division of Heart and Lungs, University Medical Centre Utrecht, Utrecht, The Netherlands

We appreciate the interest in our manuscript and the valuable comments regarding our recent publication in the Netherlands Heart Journal [1]. We fully agree with and value your important observation that digital stethoscopes, including the CORE 500, significantly benefits for cardiologists with hearing impairments. The ability of digital stethoscopes to connect via Bluetooth to modern hearing aids represents a noteworthy advancement, enhancing diagnostic clarity and enabling colleagues with hearing challenges to perform cardiovascular examinations effectively. This feature certainly deserves broader recognition among both cardiologists and audiologists.

Regarding your comment on the CE-mark, we have reached out to the manufacturer for clarification. As stated in our original article, we were unable to confirm the presence of a specific CE mark for the CORE 500 stethoscope or its accompanying app and AI features. The manufacturer informed us that their CE regulatory documentation (https://support.ekohealth.com/hc/en-us/articles/13156748696347-Regulatory) specifically applies to the 3M Littmann CORE and Eko DUO devices. Additionally, they confirmed that the CORE 500 stethoscope is currently not available for shipment to Europe; it is only available in the USA, Canada, and the UK.
